# Classification of FTLD-TDP cases into pathological subtypes using antibodies against phosphorylated and non-phosphorylated TDP43

**DOI:** 10.1186/2051-5960-1-33

**Published:** 2013-07-10

**Authors:** Rachel H Tan, Claire E Shepherd, Jillian J Kril, Heather McCann, Andrew McGeachie, Ciara McGinley, Andrew Affleck, Glenda M Halliday

**Affiliations:** Neuroscience Research Australia, Barker Street, Randwick, Sydney 2031 Australia; School of Medical Sciences, University of New South Wales, Kensington, Sydney Australia; Discipline of Pathology, Sydney Medical School, The University of Sydney, Camperdown, Sydney Australia; Discipline of Medicine, Sydney Medical School, The University of Sydney, Camperdown, Sydney Australia

**Keywords:** Pathological classification, TDP43, Frontotemporal dementia

## Abstract

**Background:**

Two commercially available TDP43 antibodies (phosphorylated or pTDP43, non-phosphorylated or iTDP43) are currently in use for the neuropathological classification of FTLD-TDP cases into pathological subtypes. To date, no studies have performed direct comparisons between these TDP43 antibodies to determine if they identify the same FTLD-TDP subtypes. The reliability of subtype classification with the use of either of these antibodies has also not been investigated. The present study compares the severity of pathological lesions identified with pTDP43 and iTDP43 in a cohort of 14 FTLD-TDP cases, and assesses the accuracy and inter-observer reliability found with either of these antibodies.

**Results:**

pTDP43 identified a greater severity of pathological inclusions across FTLD-TDP cases in comparison to iTDP43 and a higher inter-observer of subtype classification was found with this antibody.

**Conclusion:**

This study demonstrates a higher consistency across independent observers in the pathological subtyping of FTLD-TDP cases with the use of a pTDP43 antibody in comparison to the iTDP43 antibody, and corroborates the use of pTDP43 for pathological classification of FTLD-TDP cases.

## Background

**Figure 1:**
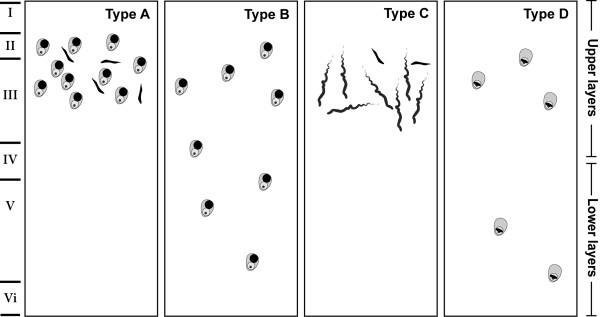
**An illustration of the harmonized classification system used for FTLD-TDP subtyping** [3]**.** Cases with moderate to numerous TDP43-immunoreactive NCI and short DN predominantly in the upper cortical layers II/III were assigned a type **A**; cases with moderate to numerous TDP43 immunoreactive NCIs and sparse DNs across all cortical layers were assigned a type **B**; cases in which long dystrophic neurites were present predominantly in the upper cortices and NCIs were assigned a type **C**; cases with numerous lentiform NII were assigned a type **D**.

TAR DNA Binding protein 43 (TDP43) is a normally occurring nuclear protein that binds RNA and DNA and plays a number of roles in transcription, RNA splicing and translational regulation [1]. The presence of abnormal TDP43 protein deposition is a pathological feature of motor neuron disease (MND) and frontotemporal lobar degeneration with TDP43-positive inclusions (FTLD-TDP) and a variety of FTLD-TDP subtypes are recognized based on the morphology and anatomical distribution of both neuronal inclusions and dystrophic neurites [2–4] (Figure 1). FTLD-TDP cases with moderate to numerous TDP43-immunoreactive neuronal cytoplasmic inclusions (NCIs) and short dystrophic neurites (DN) predominantly in the upper cortical layers II/III are designated type A (Figure 1A); cases with moderate to numerous TDP43-immunoreactive NCIs and sparse DNs across all cortical layers are designated type B (Figure 1B); cases in which long dystrophic neurites are present predominantly in the upper cortices and NCIs are rare are assigned a type C (Figure 1C); while cases with numerous neuronal intranuclear inclusions and DNs not restricted to any cortical layer are assigned a type D (Figure 1D) [3].

There are, at present, two types of commercially available TDP43 antibodies used to neuropathologically identify and classify the abnormal inclusions and neurites into the different FTLD-TDP subtypes – phosphorylated (pTDP43) and non-phosphorylated (iTDP43) TDP43 antibodies. Prior to the recent generation of pTDP43 antibody [5], iTDP43 antibodies were mainly used for this purpose with some debate on the necessity for TDP43 phosphorylation in disease pathogenesis (see [6] for a review). At present there is no consensus on the use of these different antibodies to subclassify FTLD-TDP cases [2, 7–13] and considerable differences are observed in clinicopathological association studies [14], possibly due to differences in the antibody detection method used. For example, Armstrong and colleagues [15] demonstrated an absence of distinct subtypes using iTDP43 and later went on to report that a greater number of pathological inclusions could be identified with pTDP43 [16], which is likely to influence the numbers and types of cases in different FTLD-TDP subtypes. To date there have been no direct comparisons between these types of antibodies to determine if they identify the same FTLD-TDP subtypes, particularly in C9ORF72-linked cases which are associated with added pathological heterogeneity [17, 18]. The reliability of subtype classification with the use of either of these antibodies has also not been investigated. The present study aimed to compare the severity of pathological lesions identified with two of the most commonly used pTDP43 and iTDP43 antibodies in a cohort of 14 FTLD-TDP cases, and to assess the accuracy and inter-observer reliability of these antibodies for the purpose of FTLD-TDP subtyping.

## Results

### Reliability of subtyping using pTDP43

**Figure 2:**
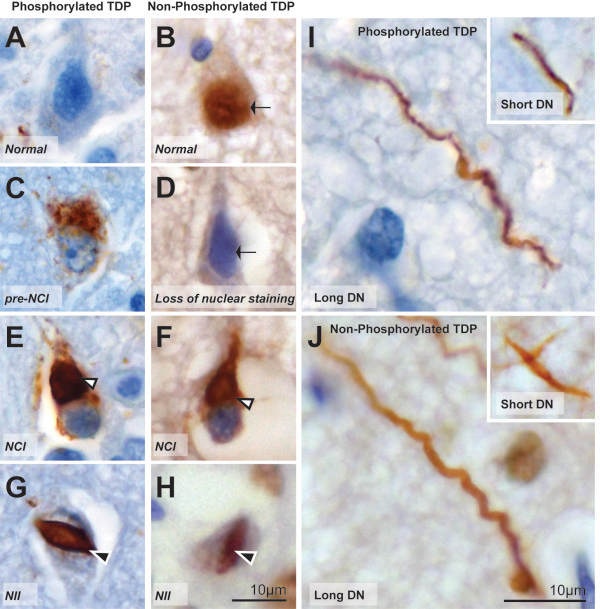
**Typical examples of TDP43-immunoreactivity with pTDP43 and iTDP43.** In sections with pTDP43, nuclear staining is not observed in normal neurons **(A)**. Typical examples of pTDP43-immunoreactive pre-inclusions **(C)**, NCI **(E)**, NII **(G)**, and long DN **(I**, inset: short DN**)**. In sections with iTDP43, normal neurons demonstrate TDP43 nuclear staining **(B)** and the loss of nuclear staining **(D)** is often accompanied by NCIs **(F)** or NIIs **(H)**. iTDP43-immunoreactive long DN **(J**, inset: short DN**)**. Scale in H (equivalent for A-G), Scale in J (equivalent for I).

**Figure 3:**
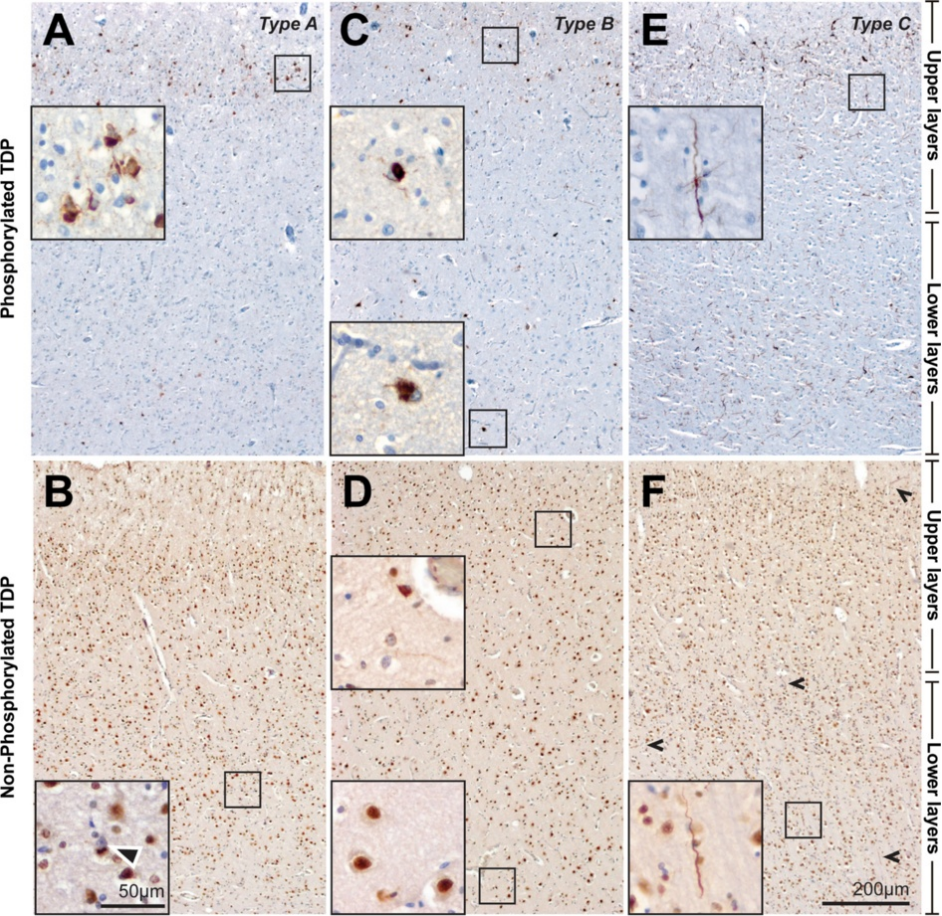
**Laminar distribution of pathological TDP43 lesions in sections of the same cases immunostained with pTDP43 (A, C, E) and iTDP43 (B, D, F).** The predominance of pathological lesions in the upper cortical layers in a case with TDP subtype A (Patient #7) was easily visualized with pTDP43 **(A)** but not with iTDP43 **(B)**. The presence of pathological lesions across all cortical layers in a case with TDP subtype B (Patient #10) was also obvious on sections with pTDP43 **(C)** but not on sections with iTDP43 **(D)**. Insets (scale=50 μm) on sections immunostained with pTDP43 **(A,** **C)** show NCIs and DNs in the upper layers of a TDP type A case **(A)** and in both the upper and lower layers in a TDP type B case **(C)**. Insets (scale=50 μm) on sections immunostained with iTDP43 **(B,** **D)** demonstrate the broad pathological overlap observed in cases with subtypes A and B – NCIs are observed in the deeper layers in a TDP type A case **(B)** but not in a TDP type B case **(D)**. While the distinctive long dystrophic neurites characteristic of a case with TDP type C (Patient #13) is obvious with both antibodies, its predominance in upper cortical layers is obvious with pTDP43 **(E)** in comparison to iTDP43 **(F**, arrows**)**.

Typical examples of the pTDP43-immunoreactive lesion pathologies are shown in Figure 2. The NCIs (Figure 2E) were rounded in shape, the NIIs (Figure 2G) lenticular or spindle-shaped, and the DNs were long and contorted or short (Figure 2I). In all cases examined, lesions were present in all cortical layers with a higher density of pathology obvious in the upper cortices of cases with subtypes A and C (Figure 3A, E). Five independent observers blind to case details determined the subtype for each case with substantial agreement (κ = 0.76). Inter-observer percentage agreement scores demonstrate difficulties were mostly in cases with subtype A (Table 1). In two cases (cases #2 and #6), a greater proportion of DNs were observed in comparison to NCIs and although long DNs were not present, a proportion of observers incorrectly classified these cases with subtype C. In another case (case #7), moderate short DNs were observed in the upper cortices whereas mild NCIs were found in all cortical layers, resulting in two observers incorrectly assigning this case a subtype B.

**Table 1:** **Inter-observer agreement for subtyping each FTLD-TDP case using sections immunostained with pTDP43 and iTDP43**

	All cases	
	pTDP43	iTDP43
TDP type A cases	74%	66%
TDP type B cases	90%	55%
TDP type C cases	93%	80%

Percentages are based on five independent ratings.

### Reliability of subtyping using iTDP43

Typical examples of the iTDP43-immunoreactive lesion pathologies are shown in Figure 2. Nuclei staining were observed in normal cells (Figure 2B). Pathological inclusions in the form of rounded NCIs (Figure 2F) and spindle-shaped NIIs (Figure 2H) usually accompanied the loss of nuclei staining in cells. Dystrophic neurites observed were long and contorted or short (Figure 2J). The laminar distribution of pathology was difficult to observe at low magnification due to the presence of nuclei staining in normal neurons, and remained difficult at higher magnifications (Figure 3B, D, F), particularly in cases with sparse pathology. Five independent observers blind to case details determined the subtype for each case with only moderate agreement using this stain (κ = 0.42). Inter-observer percentage agreement scores demonstrate difficulties in cases with subtypes A and B (Table 1). The difficulties described above with pTDP43 (cases #2, #6, #7) were also present with iTDP43, but observers noted less pathology in the iTDP43-stained sections, resulting in a larger proportion of observers incorrectly subtyping these cases. In addition to difficulties with subtype A cases, pathological lesions with iTDP43 in case #1 were very scarce and could not be subtyped by several observers. In cases #8, #9, #10 and #11 examined with iTDP43, NCIs were rare, and similar severities of DNs were seen in comparison. This resulted in a large proportion of observers assigning these TDP type B cases to subtype A.

### Comparison of pathological inclusions identified with pTDP43 and iTDP43

**Figure 4:**
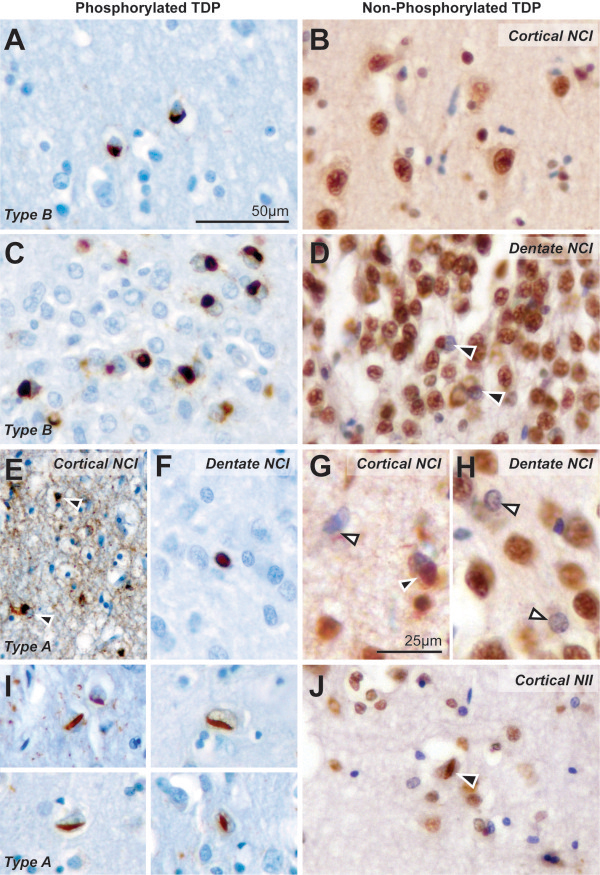
**Pathological inclusions identified in similar sections of the same cases examined with pTDP43 and iTDP43.** NCIs were observed in the cortex on sections with pTDP43 **(A)**, but not in the same region of sections with iTDP43 **(B)** in Patient #8. Moderate NCIs were observed in the dentate gyrus on sections with pTDP43 **(C)** whereas only mild NCIs were observed in the same region on sections with iTDP43 **(D**, arrows**)** in Patient #10. Compared to the NCIs observed in the upper cortices in Patient #6 with pTDP43 **(E**, black arrows**)**, iTDP43 sections demonstrated less NCIs **(G**, black arrows**)** and a loss of TDP43 nuclear staining in surrounding neurons **(G**, white arrows**)**. In Patient #1, NCIs were identified in the dentate gyrus in a case examined with pTDP43 **(F)**, but not in sections with iTDP43 **(H)** although this antibody demonstrated mild loss of nuclear staining in surrounding neurons (white arrows). NIIs were observed in the cortices of four cases (Patients #1, 3, 4, 5) with pTDP43 **(I)** but were identified only in one case (Patient #1) with iTDP43 **(J)**. Scale in **A** **(**equivalent for **B,C,D,E,F,I and J)**, Scale in **G** (equivalent for **H**).

Direct comparison between subtype classification with pTDP43 and iTDP43 showed only moderate agreement (κ = 0.58). The assessment of similar regions in the same sections of cases examined with pTDP43 and iTDP43 antibodies at high resolution is shown in Figure 4. Across all cases examined, more NCIs were identified in the cortices and dentate gyrus with pTDP43 compared to iTDP43 (Figure 4A-H). While the absence of TDP43 nuclear staining was observed in some neurons examined with iTDP43, no concomitant pathological TDP43 inclusions were present (Figure 4G, H white arrows). The finding of fewer pathological inclusions with iTDP43 is unlikely to be due to oversight, as all five observers took twice as long to examine these sections due to the staining of many normal structures. NCIs were identified in the dentate gyrus in all cases examined with pTDP43 (Figure 4C, F) but were absent from two cases examined with iTDP43, one of which demonstrated a loss of nuclear staining in surrounding neurons (Figure 4H). NIIs were sparse and observed in the cortices of four cases with pTDP43 (Figure 4I) but were identified in only one case with iTDP43 (4J). Dystrophic neurites were easily identified with both antibodies, with observers reporting similar severities in sections examined.

### Comparison of the distribution of pathology identified with pTDP43 and iTDP43

Direct comparisons of the distribution of pathology in FTLD-TDP cases on sections of the same cases immunolabelled with pTDP43 and iTDP43 are shown in Figure 3. Due to the absence of normal physiological nuclei staining with antibodies to pTDP43, pathological lesions were more obvious and as such, the distribution of TDP43 immunoreactive lesions in the cortices was easily visualised at low magnification (Figure 3A, C, E). This was not the case in sections with iTDP43 (Figure 3B, D, F). At higher magnifications (Figure 3 insets), the distribution of pathological lesions with iTDP43 in certain cases remained difficult and the most obvious reason for this being the lower pathological load observed with this type of antibody, making the predominant layers involved difficult to distinguish. Since the distinctive long dystrophic neurites characteristic of FTLD-TDP type C are easily visualized with either antibody, this subtype was easily recognised regardless of the laminar pattern of lesion pathology (Figure 3E and F). The distinction between subtypes A and B, however, is less straightforward, particularly with iTDP43. Since less pathological inclusions are identified with this antibody, there is a greater reliance on the laminar distribution to determine these subtypes. In cases examined with iTDP43, the presence of NCIs in the deeper layers of cases with subtypes A (Figure 3B), and its presence in the upper layers whilst being absent from the deeper layers in cases with subtype B (Figure 3D) contradicts the classification variables that distinguish between these subtypes, with broad pathological overlap for these subtypes using the iTDP43 antibody, as reported by others [15].

Based on the severity and distribution of pathological lesions identified, the five observers independently assigned cases to FTLD-TDP subtypes. Overall, a higher inter-observer agreement was found with typing sections stained with pTDP43 in comparison to iTDP43 across all subtypes (Table 1). Difficulties in classifying cases with iTDP43 appear to be due to the lower pathological load and the lack of an obvious cortical distribution of pathological lesions with this type of antibody, a major factor that enables the distinction between subtypes, particularly in cases where pathology is sparse.

## Discussion

This is the first study to carry out a comparison of inter-observer subtyping of TDP43 pathology using antibodies against pTDP43 and iTDP43 in the frontotemporal cortices of FTLD-TDP cases. We report here a greater inter-observer agreement with pTDP43 in comparison to iTDP43, and only a moderate agreement between the two antibodies. Direct comparisons of similar regions in the same sections of cases examined with pTDP43 and iTDP43 demonstrated more pathological inclusions with pTDP43, as well as a clearer distribution of these in the cortical sections. Overall, inter-observer reliability scores demonstrated a higher reliability and consistency in the subtyping of cases using antibodies to pTDP43.

The classification of FTLD-TDP cases into pathological subgroups is weighted on the relative severities of pathological lesions and laminar distribution of these in the cortical layers [3]. The criterion defining these subgroups was developed originally with ubiquitin immunohistochemistry, and while successfully applied to some FTLD-TDP cases, it has not been as easily recognized in a large proportion of others, most probably due to the additional pathology identified by using TDP43 immunohistochemistry [8]. In the present study, we describe further the difficulties involved in distinguishing between TDP subtypes A and B using an iTDP43 antibody. The pathological overlap between these two subtypes has been reported previously using an iTDP43 antibody [15], and we can confirm the pathological variables that underscore these observations. The lack of obvious cortical distribution of pathology largely contributed to these difficulties, particularly in cases where NCIs were rare. In comparison to using an iTDP43 antibody, both the laminar distribution of pathology and presence of cytoplasmic inclusions were more easily identified by all observers on sections stained with a pTDP43 antibody. The distinctive long dystrophic neurites characteristic of TDP type C was apparent in most instances and as such, the lack of obvious laminar patterns of pathology found with an antibody to iTDP43 did not affect the identification of this subtype. Overall, we found the inter-observer reliability scores to be higher when using a pTDP43 antibody, suggesting better consistency between the pathological lesions identified with this antibody and the current variables that define the subgroups.

While there is much debate at present with regards to phosphorylation in the process of TDP43 pathology, it has been widely accepted that the reduction in nuclear staining, as observed with an iTDP43 antibody, precedes the formation of pathological inclusion bodies (see [6] for a review). The present study notes in sections examined with an iTDP43 antibody, the lack of nuclear staining in neurons that did not have any concomitant pathological TDP43 inclusions. Together with the greater severities of inclusion bodies noted in corresponding sections with a pTDP43 antibody, this would appear to support the findings of Hasegawa and colleagues [8], demonstrating that phosphorylated TDP43 is a major component of inclusion bodies.

Of note, however, are previous reports of pTDP43 labelling intraneuronal dot-like structures that show morphologic characters consistent with granulovacuolar degeneration (GVD) [19], as well as partially co-localising with pathological tau inclusions in Alzheimer’s disease [20]. As such, while a higher accuracy in subtype classification may be found with pTDP43, iTDP43 may demonstrate higher specificity for FTLD pathologies.

## Conclusion

In summary, this study demonstrates a higher con-sistency across independent observers in the pathological subtyping of FTLD-TDP cases with the use of a pTDP43 antibody compared with an iTDP43 antibody. An added advantage of this antibody is that unlike the iTDP43 antibody, pTDP43 antibodies do not label normal physiological TDP43 in the nucleus, thereby making it easier and consequently less time-intensive to identify pathological lesions present in both the nucleus and cytoplasm of neurons, and the laminar distribution of these pathologies. Overall, the present findings corroborate the use of pTDP43 antibodies in pathological subtyping of FTLD-TDP. The higher inter-observer consistency found with this antibody will improve the strength of associations found in future clinicopathological studies performed across various laboratories using pTDP43 antibodies.

## Methods

### Cases

Fourteen cases of FTLD-TDP (6 male, 8 female, see Table 2) were selected from a clinicopathological case series collected by the Sydney Brain Bank through a regional brain donor program in Sydney, Australia. Ten relatively typical cases of TDP subtypes A, B and C as well as 4 cases with the C9ORF72 gene mutation were selected for this study. The program holds approval from the Human Ethics Committee of South Eastern Sydney and Illawarra Area Health Service and The University of New South Wales and complies with the statement on human experimentation issued by the National Health and Medical Research Council of Australia. All cases were free from coexisting neuropathologies. Diagnostic genetic [21] and neuropathological [22] screening of these cases had been conducted previously and FTLD-TDP cases previously subtyped as A (N=7, 3 with C9ORF72 gene expansions), B (N=4, 1 with C9ORF72 gene expansion) or C (N=3, without C9ORF72 gene expansions). FTLD-TDP subtype D is very rare and was not represented here nor in previous large clinicopathological studies [2, 11–13].

**Table 2:** **Demographics, clinical syndrome and pathological subtypes assigned by 5 independent raters to FTLD-TDP cases in this study**

Subtype	Case #	Age at death (y)/ Gender (M/F)	Disease duration (y)	Initial syndromes	pTDP43					iTDP43				
					**Reviewer**					**Reviewer**				
					**1**	**2**	**3**	**4**	**5**	**1**	**2**	**3**	**4**	**5**
A	1	76F	2	bvFTD	A	A	A	A	B	B	A	A	B	B
A	2	84F	8	Language FTD	C	A	A	C	C	A	A	A	C	A
A	3	54F	6	bvFTD	A	A	A	B	A	A	A	A	A	A
A	4	60M	3	Language FTD+bvFTD	A	A	A	A	A	A	A	A	A	A
A	5*	72M	9	bvFTD	A	A	A	A	A	A	A	A	C	A
A	6*	65M	2	bvFTD	A	C	A	C	A	A	C	C	B	A
A	7*	65F	3	bvFTD + MND	B	A	B	A	A	B	A	B	B	B
B	8*	55M	3	bvFTD	B	B	B	B	B	B	B	D	C	B
B	9	52F	1	bvFTD + MND	B	B	B	A	B	A	B	B	A	B
B	10	66M	2	bvFTD + MND	B	B	B	B	B	A	B	C	C	B
B	11	55F	2	bvFTD + MND	B	B	B	B	B	B	B	C	B	B
C	12	68F	1	Language FTD + MND	B	C	C	C	C	B	C	B	B	C
C	13	68M	12	bvFTD	C	C	C	C	C	C	C	C	C	C
C	14	83F	13	Language FTD	C	C	C	C	C	C	C	C	C	C

* C9ORF72 gene mutation; **bvFTD** behavioral frontotemporal dementia; **MND** motor neuron disease.

### Immunohistochemistry

Paraffin sections of 10 μm thickness were taken from the superior frontal and entorhinal cortices and hippocampus at the level of the lateral geniculate body. Immunoperoxidase staining with antibodies against pTDP43 (S409/410) (1:80,000, TIP-PTD-M01, Cosmo Bio, Tokyo Japan) was performed using the Discovery XT autostainer (Ventana Medical Systems). iTDP43 protein was visualised following microwave antigen retrieval (sections were boiled for 3 min in 0.2 M citrate buffer, pH 6.0) using TDP43 against amino acids 1–260 (1:1000; 10782-2-AP, ProteinTech Inc., Chicago, IL), peroxidase visualisation and counterstaining with 0.5% cresyl violet as described previously [23].

### Semiquantitative analysis of TDP43 immunohistochemistry

The cases were assessed by 5 experienced raters who assigned the cases to the FTLD-TDP subtypes using the recently updated classification system for FTLD-TDP pathology [3]. In each cortical region examined, abnormal TDP43-immunoreactivity was scored using semi-quantitative scoring (0 = none; + = mild; ++ = moderate; +++ = severe) [24] along strips of frontal and entorhinal cortices parallel to the pial surface, and also in the hippocampal dentate gyrus. The density of neuronal cytoplasmic inclusions (NCI) (Figure 2E, F), neuronal intranuclear inclusions (NII) (Figure 2G, H) and dystrophic neurites (DN) (Figure 2I, J) were scored separately, and for iTDP43 the absence of normal nuclear staining was also scored (Figure 2D). The distribution of pathological lesions in the cortices was also recorded (all = present in all layers; upper = restricted to upper cortices; all + upper = present in all layers with a predisposition for upper cortices). Pre-inclusions were noted (Figure 2C) but not included in the assessment of case classification. The type and distribution of pathology in the harmonized classification system was used for FTLD-TDP subtyping [3] as illustrated and detailed in Figure 1. All analyses were performed while blinded to subject details, clinical and neuropathological diagnosis.

### Statistics

The final classification of cases into pathological subtypes was determined by the most common subtype given in the present analysis using data from all observers and all stains (that were performed at the same time), and consideration of the diagnostic pathology observed during case screening (diagnostic slides performed at different times over years). The reproducibility of classifying cases into the three different subtypes (A, B and C) was tested using data from five independent researchers blind to case details and κ statistics for multiple raters [25]. For each subtype, inter-observer percentage agreement was also calculated [26] for each antibody used. Published guidelines for interpreting the κ and agreement values were used [27]: 0.00-0.20 = slight agreement, 0.21-0.40 = fair agreement, 0.41-0.60 = moderate agreement, 0.61-0.80 = substantial agreement, 0.81-1.00 = almost perfect agreement.
